# Assessment of grip strength with the modified sphygmomanometer test: association between upper limb global strength and motor function

**DOI:** 10.1590/bjpt-rbf.2014.0118

**Published:** 2015-10-06

**Authors:** Júlia C. Martins, Larissa T. Aguiar, Eliza M. Lara, Luci F. Teixeira-Salmela, Christina D. C. M. Faria

**Affiliations:** 1 Departamento de Fisioterapia, Universidade Federal de Minas Gerais (UFMG), Belo Horizonte, MG, Brazil

**Keywords:** rehabilitation, physical therapy, stroke, muscle strength, hand strength, upper extremity

## Abstract

**Background:**

Grip strength, commonly evaluated with the handgrip dynamometer, is a good indicator of upper limb (UL) function in stroke subjects and may reflect the global strength deficits of the whole paretic UL. The Modified Sphygmomanometer Test (MST) also provides objective and adequate measures at low-cost.

**Objective:**

To assess whether grip strength values obtained by using the MST and those obtained by using a handgrip dynamometer would present similar correlations with the global strength and motor function of the paretic UL in subjects with stroke, both in the subacute and chronic phases.

**Method:**

Measures of grip strength (MST and handgrip dynamometer), UL global strength (MST and hand-held dynamometer), and UL motor function (Fugl-Meyer motor assessment scale) were obtained with 33 subacute and 44 chronic stroke subjects. Pearson and Spearman correlation coefficients were calculated and Stepwise multiple regression analyses were performed to investigate predictor variables of grip strength (α=0.05).

**Results:**

Significant correlations of similar magnitude were found between measures of global strength of the paretic UL and grip strength assessed with both the MST (0.66≤*r*≤0.78) and handgrip dynamometer (0.66≤*r*≤0.78) and between UL motor function and grip strength assessed with both the MST (0.50≤*r_s_*≤0.51) and hand-held dynamometer (0.50≤*r_s_*≤0.63) in subacute and chronic stroke subjects. Only global strength remained as a significant predictor variable of grip strength for the MST (0.43≤R^2^≤0.61) and for the handgrip dynamometer (0.44≤R^2^≤0.61) for both stroke subgroups.

**Conclusion:**

Grip strength assessed with the MST could be used to report paretic UL global strength.

## Introduction

Difficulties in the use of the upper limbs (ULs) for the completion of activities of daily living are common in subjects with stroke and lead to functional limitations and participation restrictions1-4. Muscle weakness of the ULs after stroke is associated with many of these limitations4-6. Therefore, the evaluation of muscle strength is commonly performed in the rehabilitation of these subjects3,6,7. 

Grip strength, commonly evaluated with a handgrip dynamometer, has been used in both clinical practice and scientific research as an evaluative, discriminative, and predictive parameter in different population groups because it is a quick, objective, and easily implemented measure8-16. The reported reference values for this measure allow the differentiation between subjects of any age or sex10,11 and the clinical identification of muscle weakness and adequate therapeutic planning12. Grip strength shows a predictive value and relates to several factors, such as mortality and frailty (especially in elderly subjects), increased risk of falls, and functional decline^13^^-^ 16.

In subjects with stroke, grip strength is commonly affected. Moderate to high correlations between grip strength, measured with a handgrip dynamometer, and UL performance measures have been demonstrated^3^^,^ 4. In these subjects, grip strength can also be a good indicator of UL functionality and may reflect global strength deficits in the paretic UL^3^^,^ 4 ^,^ 17 ^,^ 18.

Most studies that have measured grip strength in subjects with stroke used a handgrip dynamometer^3^^,^ 4 ^,^ 18. However, in many clinical settings, the handgrip dynamometer is not available because of its relatively high cost. An alternative to measure grip strength in an objective manner and at a lower cost is to use the modified sphygmomanometer test (MST). This test is based on an adaptation of the conventional aneroid sphygmomanometer, a device commonly used and easily acquired by health professionals to measure blood pressure. It is a quick and easily implemented test that follows similar procedures to those adopted in the manual dynamometer^19^^,^ 20. In addition, a good correlation of grip strength measurements was found between the MST and the handgrip dynamometer in subjects with stroke (0.80≤*r*≤0.84)^21^ and in other population groups, such as adults and the elderly (0.75≤*r*≤0.91)^22^.

Therefore, the primary objective of this study was to assess whether grip strength values obtained with the MST and with a handgrip dynamometer would present similar correlations with the global strength and motor function of the paretic UL in subjects with stroke, both in the subacute and chronic phases. The secondary objective of this study was to determine whether the prediction model of grip strength measured with the MST would be similar to that measured with a handgrip dynamometer, both for subjects in the subacute and chronic phases of the stroke, taking into account as predictive variables those that have shown significant correlations with grip strength, including sex^10^^,^ 11, age^10^^,^ 11, UL motor function^3^^,^ 4, and global UL strength^17^^,^ 18.

## Method

### Participants

Subjects with stroke were recruited from the community and invited to participate in the study if they met the following inclusion criteria: clinical diagnosis of stroke for at least 3 months and age 20 years or older. The exclusion criteria were: cognitive impairments identified by using the Mini-Mental State Examination (cutoff point based on the school level)^23^; pain during the tests; and other neurological, rheumatologic, and/or orthopedic dysfunctions that might impair the performance of the muscle strength tests, such as rheumatoid arthritis, fractures, Parkinson's disease, multiple sclerosis, muscular dystrophy, or amyotrophic lateral sclerosis. Subjects who were unable to understand or perform the proposed tests were also excluded from the study. All of the participants signed the informed consent form approved by the research ethics committee of *Universidade Federal de Minas Gerais* (UFMG), Belo Horizonte, MG, Brazil (ETIC 0492.0.203.000-10).

The statistical software MedCalc for Windows, version 12.7.5 (MedCalc Software, Ostend, Belgium) was used to determine the number of subjects to be assessed, considering power= 0.8, *r*=0.69, and α=0.05. As a result, an *n*=14 was derived. One of the assumptions that must be met in order to use statistical tests to assess the correlation between variables is the sample variability in relation to the outcome of interest^24^. Taking into account the characteristics that could result in changes in muscle strength, the recruitment was conducted so that there was variability in relation to two age groups (20-59 years and older than 60 years) and sex (male and female). Thus, we sought to assess at least 28 subjects in this study for the subacute and chronic phases of the stroke.

### Outcome measures

Motor function of the paretic UL was assessed by using the Fugl-Meyer Motor Assessment Scale (UL section), with adequate validity and reliability in subjects with stroke^25^. The section related to UL motor function consists of 33 items, and the score for each item was added to the total score. The classification system used in this study defined the total scores as follows: 66 points, adequate motor function; between 50 and 65 points, mild motor impairment; between 30 and 49 points, moderate impairment; and <30 points, serious impairment^3^^,^ 21.

The grip strength of the paretic hand was evaluated by using a hydraulic SAEHAN^®^ grip dynamometer (Model SH5001; SAEHAN Corporation, Yangdeok-Dong, Masan, South Korea) and DuraShock^TM^Tycos^®^ aneroid sphygmomanometer (Model DS-44; Welch Allyn Inc., Skaneateles Falls, NY, USA), with the necessary adaptations to undertake the MST, as described below. The global strength of the paretic UL (wrist, elbow and shoulder flexors and extensors, and shoulder abductors) was evaluated by using both the microFET2^TM^ digital handgrip dynamometer (Hoggan Health Industries Inc., Salt Lake City, UT, USA) and the DuraShock^TM^Tycos^®^ aneroid sphygmomanometer, also with the necessary adaptations for the completion of the MST. The sphygmomanometer was adapted for the bag method. The inflatable part of the whole external Velcro that constitutes the cuff of the equipment was removed, and this structure was folded into three equal parts and placed in an inelastic cotton bag with a zipper, as described previously^19^^,^ 20.

The portable dynamometers and sphygmomanometers, purchased factory-calibrated for the completion of this study, were used as per the manufacturers' instructions. After the adaptation of the sphygmomanometer to the bag method and before using it for data collection, the recommended calibration procedures were performed^19^. The correlation between the weights (plates) and the blood pressure values (in mmHg) was high (*r=*0.99;* p≤*0.001), and the coefficient of variation ranged from 0.45% to 4.68%, without systematic error.

### Procedures

All of the tests were performed in a single day by a previously trained, single examiner. First, clinical and demographic data were collected, such as age, sex, body weight, height, post-stroke time, and type of stroke. The Fugl-Meyer Scale was applied before the muscle strength tests. Muscle strength was assessed by a single examiner, while the reading and recording of the measures were performed by another examiner. No communication was allowed between the examiners regarding the measurements obtained. Before the evaluation of muscle strength, the allocation of the equipment (portable dynamometer and modified sphygmomanometer) was performed by means of a random draw. A rest period of 2 minutes was allowed between the evaluations with the devices^26^. The measurements of global UL strength (wrist, elbow and shoulder flexors and extensors, and shoulder abductors)^3^^,^ 4 ^,^ 17 ^,^ 27 ^,^ 28 and grip strength were performed only on the paretic side, always in the same order. However, some participants were not assessed for all muscle groups because of difficulty generating the maximal isometric strength. Thus, the number of measurements for global strength of the paretic UL, considering all the muscle groups selected, was lower than the number of measurements obtained for grip strength and motor function.

**Figure 1.**
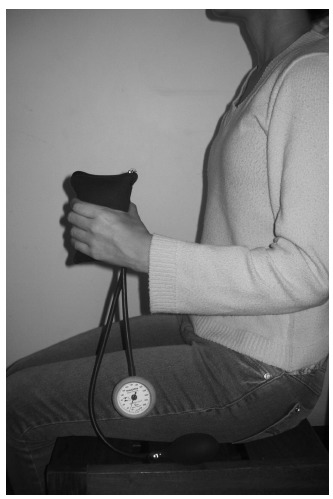
Assessment of handgrip strength with the Modified Sphygmomanometer Test.

The positions during the measurements, stabilization to prevent compensatory movements, and verbal stimulation were standardized and adopted in the measurements using the two devices. For the assessment of strength of the paretic UL by using a hand-held dynamometer and the MST, the subjects remained in the supine position, following the protocol described by Bohannon^29^ as follows: for the wrist flexors and extensors, the UL was positioned to the side of the body, with the shoulder in neutral position, the elbow flexed at 90°, manual stabilization of the forearm, and resistance applied to the distal region of the metacarpophalangeal joints. For the elbow flexors and extensors, the UL was positioned to the side of the body, with the shoulder in neutral position, the elbow flexed at 90°, manual stabilization of the shoulder, and resistance applied to the distal region of the anterior and posterior surfaces of the forearm. Meanwhile, the shoulder flexors and extensors were flexed at 90º in neutral rotation, with the elbow extended and resistance applied to the distal region of the anterior and posterior surfaces of the arm. The shoulder abductors were abducted to 46º, with the elbow extended, manual stabilization of the shoulder, and resistance applied to the distal region of the side surface of the arm. For the evaluation of grip strength, both with the handgrip dynamometer and the MST, the participants remained seated on a chair with the feet and trunk supported, shoulder adducted, elbow flexed at 90°, forearm in neutral position, wrist with 0 to 30º extension^30^ (Figure 1).

Immediately before the measurement of muscle strength, the procedures were performed for demonstration and familiarization. During the tests, the subjects were instructed to perform a maximum isometric contraction for 5 s, and the peak force was recorded. The volunteers received verbal stimulus to start the movement and maintain contraction as follows: "Ready, set, GO! ... Harder! ... Harder! ... Harder! ... That's it! ... Relax!"^31^. Only one trial was performed after the familiarization because, according to a previous study^32^, strength did not differ between the first trial, average of the first two trials, and average of three trials. If the examiner recognized some compensatory movement by the participant, a new measurement was obtained and registered^21^^,^ 32. The force exerted on the modified sphygmomanometer was determined by pressure gauge reading, considering increments of 2 mmHg. Before each measurement, the examiner ensured that the equipment was pre-inflated at 20 mmHg^33^.

### Statistical analysis

Descriptive statistics and normality tests (Shapiro-Wilk) were performed. Normally distributed data were observed for all continuous variables. The global strength of the paretic UL was calculated (sum of the muscle strength values of the flexors and extensors of the wrist, elbow and shoulder, and shoulder abductors) for the portable dynamometer and MST measurements. The Pearson correlation coefficient was calculated to determine whether the grip strength values obtained by using the portable dynamometer and those obtained by using the MST correlated with the global strength of the paretic UL, which was also measured by using the two devices. The Spearman correlation coefficient was used to investigate the correlation between the grip strength values and the motor function of the paretic UL. When the coefficient values were statistically significant, the magnitudes of the correlations were classified as follows^34^: very low, ≤0.25; low, 0.26-0.49; moderate, 0.50-0.69; high, 0.70-0.89; and very high, 0.90-1.00. To investigate the predictive relationship between grip strength and the global strength and motor function of the paretic UL, four models of stepwise multiple regression were analyzed, two for the subjects with stroke in the subacute phase and two for the subjects with stroke in the chronic phase. For each of the samples, the dependent variable was the grip strength of the paretic hand, evaluated by using a dynamometer, in one model and muscle strength, assessed by using the MST, in another model. 

 / The predictive variables of all the models were sex, age, UL motor function, and global strength of the paretic UL (variables already pointed out by previous studies as having significant relationships with grip strength). It is worth noting that, for the model where the dependent variable was grip strength evaluated by using the MST, the global strength of the paretic UL, also measured by using the MST, was considered as a predictive variable, maintaining similarities with the portable dynamometer measurements. The statistical assumptions for the regression analysis were checked and adequately met (α=5%).

## Results

Altogether, 33 subjects were evaluated in the subacute phase (17 men; mean age, 62±12 years; time post stroke, 3.8±0.7 months) and 44 in the chronic phase (24 men; mean age, 60±15 years; time post stroke, 80±77 months). The clinical and demographic characteristics of these subjects are described in Table 1. Table 2 presents the results of the descriptive statistics of the measurements of muscle strength and motor function of the paretic UL for the subacute and chronic groups.

**Table 1.** Subjects' demographic and clinical characteristics.

**Characteristics**	**Subacute (****n***=33)***	**Chronic (****n***=44)***
Age (years): mean (SD); range [min-max]	62 (12); [29-85]	60 (15); [25-86]
Time since the onset of stroke (months): mean (SD); range [min-max]	3.8 (0.74) [3-5]	80 (77); [6-371]
Body mass index (kg/m^2)^: mean (SD)	25 (5)	26 (4)
Gender: Men, n (%)	17 (52%)	24 (55%)
Paretic side: right, n (%)	18 (55%)	21 (48%)
Type of stroke, n (%)		
Ischemic	30 (91%)	35 (80%)
Hemorrhagic	3 (9%)	4 (9%)
Both ischemic and hemorrhagic	0	5 (11%)
Fugl Meyer - Upper extremity section (0-66)*, n* (%)		
Mild motor impairments (66-50)	32 (97%)	31 (71%)
Moderate motor impairments (49-30)	1 (3%)	8 (18%)
Severe motor impairments (<30)	0	5 (11%)

SD: Standard Deviation.

**Table 2.** Descriptive statistics regarding the measures of handgrip strength, global strength, and motor function of the paretic upper limb.

**Measure**	**Devices**	**n **	**Mean (SD)**	**95% CI**	**Range [min-max]**
**Subacute phase**					
Handgrip strength	Modified sphygmomanometer (mmHg)	33	175 (78)	147-202	54-290
	Handgrip dynamometer (kg)	33	21 (10)	18-25	6-40
Global UL strength	Modified sphygmomanometer (mmHg)	27	1021 (232)	930-1113	516-1,382
	Hand-held dynamometer (kg)	27	75 (20)	68-83	36-108
UL motor function	Fugl-Meyer scale (0-66 points)	33	61 (5)	60-63	48-66
**Chronic phase**					
Handgrip strength	Modified sphygmomanometer (mmHg)	44	142 (64)	123-161	50-304
	Handgrip dynamometer (kg)	44	18 (9)	15-21	4-40
Global UL strength	Modified sphygmomanometer (mmHg)	36	807 (248)	723-803	402-1,308
	Hand-held dynamometer(kg)	36	58 (24)	50-67	17-123
UL motor function	Fugl-Meyer scale (0-66 points)	44	52 (15)	47-56	6-66

UL: Upper limb; SD: Standard deviation; CI: Confidence interval.

As shown in Table 3, grip strength showed significant moderate to high correlations with the measurements of motor function and global strength of the paretic UL, both for the MST values (0.50≤*r_s_*≤0.51 and 0.66≤*r_s_*≤0.78, respectively) and the hand-held dynamometer (0.50≤*r_s_*≤0.63 and 0.66≤*r_s_*≤ 0.78, respectively). 

**Table 3.** Correlation coefficients between the measurements of handgrip strength assessed with both devices and variables related to global strength and motor function of the paretic upper limb.

**Variables**	**N**	**Handgrip strength**	**n**	**Handgrip strength**
		**(MST)**		**(Handgrip dynamometer)**
Subacute phase				
UL global strength	27	*r*=0.78; *p*<0.0001	27	*r*=0.78; *p*<0.0001
UL motor function	33	*r_s_*=0.50; *p*=0.006	33	*r_s_*=0.50; *p*=0.003
**Chronic phase**				
UL global strength	36	*r*=0.66; *p*<0.0001	36	*r*=0.66; *p*<0.0001
UL motor function	44	*r_s_*=0.51; *p*<0.0001	44	*r_s_* =0.63; *p*<0.0001

MST: Modified Sphygmomanometer Test; UL: Upper Limb; r_s_:Spearman correlation coefficient; r: Pearson correlation coefficient.

As can be seen in Table 4, both for the subjects in the subacute and chronic phases of the stroke, the regression model for the measurements obtained with the MST presented similar results to those for measurements obtained with a handgrip dynamometer. Among the four predictive variables considered for each of the four models, only one was retained in all of them. The global strength of the paretic UL had significant predictive values ranging from 0.43 to 0.61 (*p*<0.001; Table 4).

**Table 4.** Results of each of the four regression models (Stepwise Method†).

**Predictor Variable**	**Dependent Variable**						**Dependent Variable**					
**Model**	**Handgrip strength (MST)**						**Handgrip strength (Handgrip dynamometer)**					
Subacute phase												
	**B**	**SE**	**β**	**R** ^**2**^	**F**	**p**	**B**	**SE**	**β**	**R** ^**2**^	**F**	**p**
Paretic UL global strength	0.25	0.04	0.78	0.61	38.78	<0.001	0.38	0.06	0.78	0.61	38.85	<0.001
Chronic phase												
Paretic UL global strength	0.17	0.03	0.65	0.43	25.91	<0.001	0.25	0.05	0.66	0.44	26.73	<0.001

*†* All of the Stepwise regression models had as input variables: sex, age, motor function of upper limb, and global strength of the paretic upper limb. MST: Modified Sphygmomanometer Test; UL: Upper Limb; *B*: Regression Coefficient; *SE*: Standard error of regression coefficient; β: standardized regression coefficient; *R* 2: Coefficient of determination.

## Discussion

The present study demonstrated that the grip strength values obtained with the MST presented a significant positive correlation and similar magnitude (moderate to high) with those obtained by using a handgrip dynamometer for both global strength and motor function of the paretic UL in the subjects in the subacute and chronic phases of the stroke. In addition, the predictive model of grip strength measured with the MST was similar to that measured with a handgrip dynamometer both for subjects in the subacute and chronic phases. Among the four variables considered as possible predictors (sex, age, UL motor function, and global UL strength), only global UL strength presented a significant predictive value for grip strength.

The similarity of the statistical results, considering the measurements obtained with the MST and a handgrip dynamometer, reinforce the positive results that have been pointed out for the validity of the MST^20^^,^ 21. Therefore, in addition to the appropriate test-retest and interrater reliability and adequacy of the concurrent validity for the assessment of muscle strength of subjects with stroke^20^^,^ 21, the grip strength values obtained by using the MST can also be used for a better understanding of the motor characteristics of the paretic UL of these subjects, both in the subacute and chronic phases, which reinforces its validity and applicability.

Investigation of the correlation between grip strength and UL performance test has been recommended for a better understanding of motor recovery of the UL of subjects with stroke^3^^,^ 35. Previous studies that examined the correlation between grip strength, measured with a handgrip dynamometer, and UL motor function in subjects with stroke reported significant moderate to very high correlations in various clinical tests of UL functions as follows: the Fugl-Meyer Scale (0.69≤*r*≤0.84)^18^^,^ 35, box and block test (0.69≤*r*≤0.97)^3^^,^ 18 ^,^ 36, nine-hole peg test (0.54≤*r*≤0.79)^3^^,^ 36, finger-to-nose test (0.69≤*r*≤0,90)^18^^,^ 35, upper extremity performance test for the elderly (TEMPA; 0.82≤*r*≤0.90)^3^^,^ 18, motor activity log (MAL; *r*=0.61)^4^, Chedoke arm and hand activity inventory (*r*=0.69)^4^, motricity index (0.83≤*r*≤0.87)^36^, Frenchay arm test (0.86≤*r*≤0.90)^36^, and motor club assessment (0.81≤*r*≤0.86)^36^.

Despite the previously reported significant and positive correlations between grip strength and UL motor function^3^^,^ 4 ^,^ 18 ^,^ 35 ^,^ 36 and even those found in this study, as we considered a multiple regression model along with other variables (sex, age, and global strength of the paretic UL), motor function showed no significant predictive power for grip strength. In fact, among the four variables considered as possible predictors, only the global strength of the paretic UL was retained in the models analyzed, both in the subjects in the subacute and chronic phases of the stroke. Studies that assessed motor function by using the Fugl-Meyer Scale and correlated it with grip strength^18^^,^ 35, assessed by using a handgrip dynamometer in subjects with stroke, did not perform a multiple regression analysis similar to that of the present study. In one of these studies^18^, a simple regression analysis was performed (*r* 2=0.71/linear regression and *r* 2=0.72/quadratic regression). The results of the present study indicate that the motor function assessed by using the Fugl-Meyer Scale was not a good predictor of grip strength when considered in conjunction with the global UL strength. The important predictive power of global strength of the paretic UL associated with the fact that the Fugl-Meyer Scale does not include manual tasks that require great grip strength may be a possible explanation of this result.

In the present study, moderate to high correlations were found between grip strength and the global strength of the paretic UL measured with a portable dynamometer and the MST in both subgroups. In addition, the global UL strength was the only predictor of grip strength in all of the models analyzed (the MST and portable dynamometer for subjects in the subacute and chronic phases of the stroke). In a systematic review, which included healthy subjects and subjects with several health conditions (stroke, amyotrophic lateral sclerosis, and Duchenne muscular dystrophy), it was concluded that the measurement of only one muscle group may be sufficient to characterize the global strength of the limb tested^37^. A study^27^ conducted with subjects with different health conditions, including stroke, fractures, and pneumonia, showed a correlation between grip strength, measured with a handgrip dynamometer, and global UL strength (sum of the values of elbow flexor strength, shoulder abductor strength, and grip strength), measured with the manual muscle test (0.70≤*r*_s_≤0.75_)._ Studies with subjects in the acute post-CVA phase^17^^,^ 28 observed a high correlation (0.73≤*r*≤0.86) between grip strength and UL strength (sum of the values of elbow flexor and shoulder abductor strengths), both measured by using a portable dynamometer. According to the authors of these studies^17^^,^ 28, grip strength obtained with a handgrip dynamometer is a good indicator of the muscle strength of subjects with stroke, having the advantage of being fast and easy to measure. Considering the results of the present study, the same can be said for grip strength obtained by using both a handgrip dynamometer and the MST. The global strength of the paretic UL is a strong and isolated predictor of grip strength even when considering variables that already displayed significant correlation with grip strength (e.g. sex, age, and motor function).

A limitation of this study was the small number of participants with high degrees of disability. Another limitation was the presence of an independent examiner to read and record the measurements. Although this is not common in clinical practice, this procedure was adopted to ensure the internal validity of the study by blinding the principal examiner of the results obtained with each of the devices. The scale selected for the evaluation of UL motor function can also be singled out as a limitation of the study, as UL functionality involves various tasks^3^^,^ 38 not covered in the scale adopted. However, we chose to use the Fugl-Meyer Scale to assess motor function of the paretic UL because it is widely used in clinical practice and research and has been used to investigate the correlation with grip strength measured with a handgrip dynamometer. Therefore, further studies are required to investigate the correlation between grip strength values obtained with the MST and those obtained with other tests, such as the box and block test^3^^,^ 18 ^,^ 36, nine-hole peg test^3^^,^ 36, finger-to-nose test^18^^,^ 35, TEMPA^3^^,^ 18, and MAL^4^, which better evaluate UL motor function and have been studied for their correlation with the handgrip dynamometer.

In conclusion, grip strength values obtained with the MST presented a significant positive correlation and similar magnitude (moderate to high) with those obtained by using a handgrip dynamometer for both global strength and motor function of the paretic UL in subjects in the subacute and chronic phases of stroke. However, only global UL strength was retained in the predictive model of grip strength. The results of the models were significant and similar to those obtained by using a handgrip dynamometer and the MST in these subjects. Therefore, we recommend measuring grip strength with the MST in subjects in the subacute and chronic phases of stroke to establish a predictive relationship with global strength of the paretic UL, as the MST provides objective and low-cost measurements, is accessible, and has presented similar statistical results to those obtained with a manual dynamometer. Further studies are necessary to assess the correlation of grip strength obtained with the MST with other outcomes, and for discriminative, predictive, and evaluative purposes.
